# The relationship between serology of hepatitis E virus with liver and kidney function in kidney transplant patients

**DOI:** 10.17179/excli2016-232

**Published:** 2016-06-02

**Authors:** Abbas Ali Zeraati, Fatemeh Nazemian, Ladan Takalloo, Amirhossein Sahebkar, Elahe Heidari, Mohammad Ali Yaghoubi

**Affiliations:** 1 Kidney transplantation Complications Research Center, Mashhad University of Medical Sciences, Mashhad, Iran; 2 Biotechnology Research Center, Mashhad University of Medical Sciences, Mashhad, Iran; 3 Metabolic Research Centre, Royal Perth Hospital, School of Medicine and Pharmacology, University of Western Australia, Perth, Australia; 4 Department of Pediatrics, Imam Reza Hospital , Mashhad University of Medical Sciences, Mashhad, Iran; 5 Endocrine Research Center, Ghaem Hospital, Mashhad University of Medical Science, Mashhad, Iran

**Keywords:** hepatitis E, kidney transplant, creatinine, alanine aminotransferase, aspartate aminotransferase, alkaline phosphatase

## Abstract

Although hepatitis E virus (HEV) is well known to cause acute hepatitis, there are reports showing that HEV may also be responsible for progression of acute to chronic hepatitis and liver cirrhosis in patients receiving organ transplantation. In this study, we aimed to evaluate the prevalence of HEV in patients with kidney transplantation. In this study, 110 patients with kidney transplantation were recruited, and anti-HEV IgG, creatinine, alanine transaminase (ALT), aspartate aminotransferase (AST), alkaline phosphatase (ALP), and estimated glomerular filtration rate (eGFR) in the first, third and sixth months after renal transplantation were measured. The mean serum anti-HEV IgG titers in the study participants was 1.36 (range 0.23 to 6.3). Twenty-three patients were found to be seropositive for HEV Ab defined as anti-HEV IgG titer > 1.1. The difference in liver and renal function tests (creatinine, eGFR, AST, ALT and ALP) at different intervals was not significant between patients with HEV Ab titers higher and lower than 1.1 (p > 0.05). However, an inverse correlation was observed between HEV Ab and eGFR values in the first (p = 0.047, r = -0.21), third (p = 0.04, r = -0.20) and sixth (p = 0.04, r = -0.22) months after renal transplantation in patients with HEV Ab < 1.1 but not in the subgroup with HEV Ab > 1.1. Also, a significant correlation between age and HEV Ab levels was found in the entire study population (p = 0.001, r = 0.33). Our findings showed a high prevalence of seropositivity for anti-HEV IgG in patients receiving renal transplants. However, liver and renal functions were not found to be significantly different seropositive and seronegative patients by up to 6 months post-transplantation.

## Introduction

Hepatitis E, which is generally a self-limiting disease, is caused by hepatitis E virus (HEV) (Balayan et al., 1983[7]; Gupta and Smetana, 1957[12]). This virus is a single-stranded RNA virus without envelope, and is the only member of hepevirus from the Hepeviridae family (Kane et al., 1984[18]; Koonin et al., 1992[24]). Distribution of HEV, which was previously known as non-A and non-B hepatitis, is mainly through water and feces, as is for viral hepatitis A (HAV) (Balayan et al., 1983[7]; Khuroo, 1980[21]). However, the transmission of HAV is much easier with global distribution and causes more infections whereas HEV is more distributed in industrialized countries (Clemente-Casares et al., 2003[10]; Tang et al., 1991[35]). The highest incidence of HEV infection is reported in Asia, Africa, the Middle East and Central America (Arankalle et al., 1988[5]; Gupta and Smetana, 1957[12]). Iran is one of the countries where hepatitis E is endemic, and several HEV outbreaks have occurred so far (Alavian et al., 2009[3]; Ghorbani et al., 2007[11]). Although the transmission of hepatitis E virus has been reported from animal to human in developed countries, the major route of transmission is through the fecal-oral route. It has also been found that the consumption of undercooked meat or meat products is a major risk factor for HEV infection (Legrand-Abravanel et al., 2010[26]). The most important diagnostic methods of HEV are PCR amplification of virus RNA or detection of Immunoglobulin M (IgM) antibodies against HEV. Currently, serological tests and qualitative and quantitative assessment of HEV RNA are considered as the gold standard tests for detection of HEV both for diagnostic and epidemiological purposes (Baylis et al., 2011[8]; Khudyakov and Kamili, 2011[19]; Takahashi et al., 2005[34]). Several interventions such as elimination of chronic infection after reduction or discontinuation of immunosuppressive therapy, use of oral ribavirin and pegylated interferon have been suggested to be effective in the management of HEV infection. However, there is as yet no proven treatment for acute or chronic hepatitis E in organ transplant patients (Aggarwal and Jameel, 2011[2]; Chaillon et al., 2011[9]; Kamar et al., 2011[15], 2014[16]). 

Hepatitis E is responsible for acute hepatitis, and it was previously believed that HEV cannot be developed to chronic hepatitis. However, more recent reports show that HEV can lead to chronic hepatitis as well as liver cirrhosis in patients receiving organ transplants (Aggarwal, 2008[1]; Koning et al., 2015[23]). It has been proposed that HEV does not usually lead to chronic hepatitis, except in patients who have solid organ transplantation or those whose immune system is suppressed (Khuroo et al., 1980[22]). Patients with organ transplantation are more susceptible to diseases caused by rare infectious viruses such as HEV; thus, differential diagnosis of pathogens such as hepatitis E virus in transplant recipients is suggested to be applied according to the reports showing that there is a relatively rapid progression to cirrhosis and chronic hepatitis E in people who have had organ transplants (Ibarra et al., 1994[14]; Stefanidis et al., 2004[32]). Seroprevalence studies have shown that 6 to 15.6 % of kidney transplant patients are positive for anti-HEV IgG. In addition, studies have shown that HEV infection is commonly observed in patients with transplantation of solid organs such as the kidneys, liver and pancreas (Kamar et al., 2008[17]; Khuroo, 2008[20]). The frequency of acute HEV infection in French patients with solid organ transplantation has been reported to be between 5 and 6.5 % (Legrand-Abravanel et al., 2011[27]). Other factors that may lead to chronic HEV infection in patients with kidney transplantation include low lymphocyte (CD2, CD3 and CD4) count, low platelet count, young age < 52 years and lymphopenia (Kamar et al., 2011[15]; Legrand-Abravanel et al., 2011[27]).

Some studies have investigated the prevalence of hepatitis E in patients with kidney transplantation (Kamar et al., 2008[17]; Legrand-Abravanel et al., 2011[27]). Given the high prevalence of transaminitis in the early stages after kidney transplantation and also uncertainty about the role of hepatitis E, in this study, the serology of hepatitis E was evaluated in relation to hepatic and renal function indices in a group of patients with kidney transplantation.

## Methods

### Data collection and population size calculation

Demographic information of patients including age, sex, and type of organ transplant donor (living or cadaver) was collected based on medical history and questionnaire. All patients underwent standard triple immunosuppressive therapy with prednisone, cyclosporine and Mycophenolate mofetil. Samples were randomly selected from patients with kidney transplantation in the Montaserieh Hospital, Mashhad, Iran. Patients with transplant rejection or positive hepatitis B surface antigen (HbsAg), anti-hepatitis C virus (HCV), and anti-cytomegalovirus (CMV) IgM were excluded. Considering that the prevalence of HEV infection has been reported to be 7.7 % in previous studies (Kamar et al., 2008[17]; Khuroo, 2008[20]; Legrand-Abravanel et al., 2011[27]), the population size was calculated to be 110 patients using the following formula.

N = (1.96)^2^×p(1-p)/d^2^=(1.96)^2^×0.077(1-0.077)/(0.05)^2^=110

### Biochemical assessment

Serum levels of anti HEV IgG, creatinine, alanine aminotransferase (ALT), aspartate aminotransferase (AST), alkaline phosphatase (ALP), and estimated glomerular filtration rate (eGER) were measured at the first week, first month, third month, and sixth month post-transplantion. To determine anti-HEV IgG level, 2 mL of venous blood were taken and measured with ELISA after separating the serum from the blood. eGFR was calculated by the modification of diet in renal disease (MDRD) using the following equation:

MDRD = 175.0 × (Serum Creatinine)^-1.154^

× (Age)^-0.203^ × 0.742 (if female) 

× 1.21 (if black)

### Statistical analysis

Descriptive data are expressed as mean ± SD. Between-group comparisons were performed using independent samples *t*-test (for normally distributed data) or Mann-Whitney U (for non-normally distributed data) test. Pearson (for normally distributed data) or Spearman (for non-normally distributed data) correlation coefficients test were used to evaluate the correlation between serology of hepatitis E with renal and hepatic function tests. All statistical analyses were performed using SPSS 15 software, and p-values less than 0.05 were considered as statistically significant.

## Results

### Demographic information of the patients

Among patients with renal transplantation admitted to the Montaserieh hospital, 110 patients were randomly selected. The patients were enrolled after obtaining written informed consent. Of the enrolled participants, 59 patients (64.53 %) were male and 51 (36.46 %) were female. The mean age of patients was 36.97 years, ranging between 15 and 62 years. 

### Assessment of biochemical markers

Most of the patients (93.6 %) had hemodialysis before receiving the transplanted kidney, and 35 patients had a history of blood transfusion (31.82 %). The mean serum anti-HEV IgG titers in the study participants was 1.36 (range 0.23 to 6.3). Antibody titer for anti-HEV IgG was positive for 23 patients (20.9 %), and negative in 86 (78.18 %); in addition, antibody levels in 1 case was on the borderline (0.91 %) that was further considered as seronegative.

The results showed that there is no significant difference in serum concentrations of creatinine, AST, ALT, and ALP at different intervals between patients with HEV Ab titers higher and lower than 1.1. The mean value of serum ALP six months after transplantation in seropositive and seronegative groups was 91.105 ± 69.251 and 57.237 ± 85.101, respectively, that was not statistically significant (p = 0.55). Mean serum creatinine level at the sixth month after transplantation in the seropositive and seronegative groups were 1.52 ± 0.48 and 1.42 ± 0.39, respectively, indicating that the difference between seropositive and seronegative groups was not significant (p = 0.33). In the group of patients with HEV Ab lower than 1.1, a negative correlation was found between the HEV Ab and the eGFR in the first month (p = 0.047, r = -0.21), the third month (p = 0.04, r = -0.2) and the sixth month (p = 0.04, r = -0.22) after transplantation. However, no significant difference was observed in serum concentrations of creatinine, AST, ALT, and ALP at different intervals between patients with HEV Ab titers higher and lower than 1.1. Moreover, a positive correlation was found between age and serum HCV Ab titers (p = 0.001, r = 0.33) in the entire study population. Serum concentrations of creatinine, AST, ALT, ALP and eGFR at different intervals in the seropositive and seronegative subjects are demonstrated in Table 1(Tab. 1).

Raw data are shown in Supplementary Table 1.

## Discussion

Kidney transplant recipients are known to be more susceptible to viral infections and show more severe clinical manifestation compared with healthy individuals (Quintana et al., 2005[30]; Tang et al., 1991[35]). More susceptibility to viral infections in these patients is mainly due to immunosuppression caused by immunosuppressive drugs. Although hepatitis E was once thought to be a self-limited acute infection that rarely leads to chronic infection, several recent investigations have reported chronic hepatitis and even cirrhosis related to hepatitis E in organ transplant recipients (Ibarra et al., 1994[14]; Tsega et al., 1991[37]; Yamashita et al., 2009[38]). 

In a study in 2011, it was shown that there was no significant difference in serum ALT levels between patients with positive and negative anti-HEV IgG (Rostamzadeh Khameneh et al., 2011[31]). It was also shown that there is no association between hepatitis E infection and increased serum levels of ALT. Hence, it was deduced that anti-HEV IgG antibodies is significantly higher in kidney transplant patients compared with general or hemodialysis population. In the mentioned study, of the total number of patients, 28 patients were seropositive (30.8 %), whereas in our study, the prevalence of seropositivity for anti-HEV IgG was 20 %. Moreover, consistent with the above-mentioned study, our findings showed that there was no significant association between serum ALT levels and anti-HEV IgG in seropositive and seronegative groups. Nonetheless, some studies have reported a positive association between the seroprevalence of HEV and blood transfusion in patients with organ transplantation (Arankalle et al., 1995[6]; Lynch et al., 1995[29]; Sylvan, 1998[33]). In a previous study on the epidemiology of anti-HEV IgG antibodies in healthy blood donors of Iran, the prevalence of HEV infections was reported to be 7.7 % (Arabzadeh et al., 2010[4]; Hesamizadeh et al., 2016[13]). It has also been shown that in a particular group of patients, such as patients undergoing hemodialysis, HEV seroprevalence correlates with the prevalence of infections in the community (Taremi et al., 2008[36]). In different populations receiving kidney or liver transplantation, the HEV seroprevalence patients were reported between 1 % and 14.5 % (Koonin et al., 1992[24]; Yamashita et al., 2009[38]). Outbreak of hepatitis E also varies in different geographical areas. According to epidemiological studies, low prevalence of anti-HEV IgG antibodies have been reported in countries such as Israel (8.2 %) and Turkey 8.3 %, but the prevalence of antibodies is rather high in countries such as Iraq (14.8 %), Saudi Arabia (16.4 %) and Pakistan (17.5 %) (Kane et al., 1984[18]; Krawczynski and Bradley, 1989[25]; Yamashita et al., 2009[38]). In a study that was conducted on 700 patients with organ transplantation, 34 patients were found to be infected with HEV one year after transplantation, and among them, 47 % were identified with chronic HEV and accordingly the annual incidence of HEV was reported to be 3.2 % (Lu et al., 2006[28]). 

In conclusion, our findings in this study indicated that the prevalence of anti-HEV IgG is rather high (20 %) in Iranian patients with renal transplantation. However, it was shown that serum levels of creatinine, AST, ALT, and ALP, and eGFR at different intervals is comparable between patients with HEV Ab titers higher and lower than 1.1.

## Acknowledgement

The authors are thankful to the Research Council of the Mashhad University of Medical Sciences for the financial support of this study. 

## Conflict of interest

The authors declare no conflict of interest.

## Supplementary Material

Supplementary table 1

## Figures and Tables

**Table 1:**
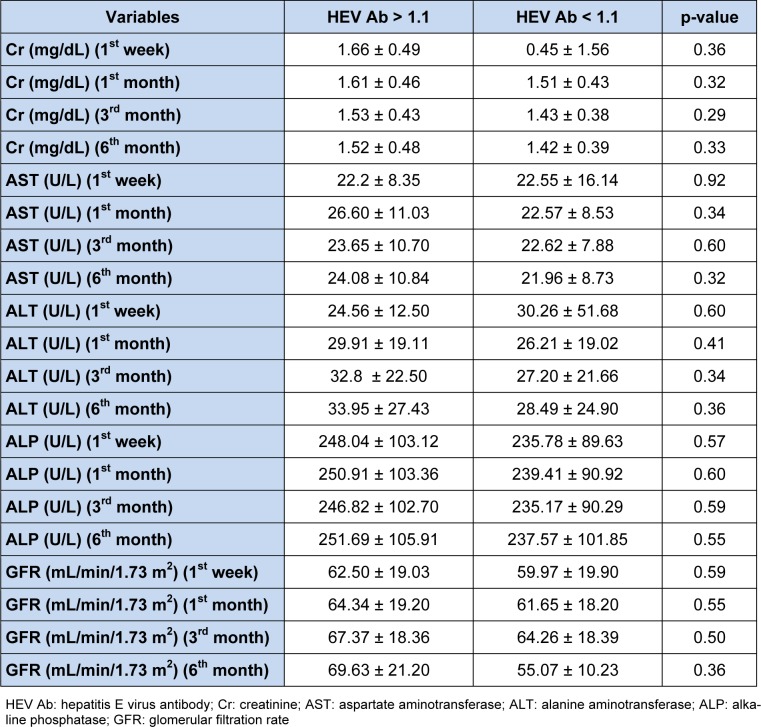
Values of renal and hepatic function indices at different intervals post-transplantation

## References

[1] Aggarwal R (2008). Hepatitis E: does it cause chronic hepatitis?. Hepatology.

[2] Aggarwal R, Jameel S (2011). Hepatitis E. Hepatology.

[3] Alavian SM, Fallahian F, Lankarani KB (2009). Epidemiology of Hepatitis E in Iran and Pakistan. Hepat Mon.

[4] Arabzadeh S, Zahedi M, Mollaei H, Aghaie-Afshar A (2010). Seroepidemiology of Anti-HEV IgG in healthy men blood donors in Kerman, 2007-2008. Iran J Virol.

[5] Arankalle VA, Chadha MS, Mehendale SM, Banerjee K (1988). Outbreak of enterically transmitted non-A, non-B hepatitis among schoolchildren. Lancet.

[6] Arankalle VA, Tsarev SA, Chadha MS, Alling DW, Emerson SU, Banerjee K (1995). Age-specific prevalence of antibodies to hepatitis A and E viruses in Pune, India, 1982 and 1992. J Infect Dis.

[7] Balayan MS, Andjaparidze AG, Savinskaya SS, Ketiladze ES, Braginsky DM, Savinov AP (1983). Evidence for a virus in non-A, non-B hepatitis transmitted via the fecal-oral route. Intervirology.

[8] Baylis SA, Hanschmann KM, Blumel J, Nubling CM (2011). Standardization of hepatitis E virus (HEV) nucleic acid amplification technique-based assays: an initial study to evaluate a panel of HEV strains and investigate laboratory performance. J Clin Microbiol.

[9] Chaillon A, Sirinelli A, De Muret A, Nicand E, d'Alteroche L, Goudeau A (2011). Sustained virologic response with ribavirin in chronic hepatitis E virus infection in heart transplantation. J Heart Lung Transplant.

[10] Clemente-Casares P, Pina S, Buti M, Jardi R, Martin M, Bofill-Mas S (2003). Hepatitis E virus epidemiology in industrialized countries. Emerg Infect Dis.

[11] Ghorbani GA, Alavian SM, Esfahani AA, Assari S (2007). Seroepidemiology of hepatitis E virus in Iranian soldiers. Hepat Mon.

[12] Gupta D, Smetana H (1957). The histopathology of viral hepatitis as seen in the Delhi epidemic (1955-56). Indian J Med Res.

[13] Hesamizadeh K, Sharafi H, Keyvani H, Alavian SM, Najafi-Tireh Shabankareh A, Sharifi Olyaie R (2016). Hepatitis A virus and Hepatitis E virus seroprevalence among blood donors in Tehran, Iran. Hepat Mon.

[14] Ibarra HV, Riedemann SG, Siegel FG, Reinhardt GV, Toledo CA, Frosner G (1994). Hepatitis E virus in Chile. Lancet.

[15] Kamar N, Garrouste C, Haagsma EB, Garrigue V, Pischke S, Chauvet C (2011). Factors associated with chronic hepatitis in patients with hepatitis E virus infection who have received solid organ transplants. Gastroenterology.

[16] Kamar N, Izopet J, Tripon S, Bismuth M, Hillaire S, Dumortier J (2014). Ribavirin for chronic hepatitis E virus infection in transplant recipients. N Engl J Med.

[17] Kamar N, Selves J, Mansuy JM, Ouezzani L, Peron JM, Guitard J (2008). Hepatitis E virus and chronic hepatitis in organ-transplant recipients. N Engl J Med.

[18] Kane MA, Bradley DW, Shrestha SM, Maynard JE, Cook EH, Mishra RP (1984). Epidemic non-A, non-B hepatitis in Nepal. Recovery of a possible etiologic agent and transmission studies in marmosets. JAMA.

[19] Khudyakov Y, Kamili S (2011). Serological diagnostics of hepatitis E virus infection. Virus Res.

[20] Khuroo MS (2008). Hepatitis E virus. Curr Opin Infect Dis.

[21] Khuroo MS (1980). Study of an epidemic of non-A, non-B hepatitis. Possibility of another human hepatitis virus distinct from post-transfusion non-A, non-B type. Am J Med.

[22] Khuroo MS, Saleem M, Teli MR, Sofi MA (1980). Failure to detect chronic liver disease after epidemic non-A, non-B hepatitis. Lancet.

[23] Koning L, Charlton MR, Pas SD, Heimbach JK, Osterhaus AD, Watt KD (2015). Prevalence and clinical consequences of Hepatitis E in patients who underwent liver transplantation for chronic Hepatitis C in the United States. BMC Infect Dis.

[24] Koonin EV, Gorbalenya AE, Purdy MA, Rozanov MN, Reyes GR, Bradley DW (1992). Computer-assisted assignment of functional domains in the nonstructural polyprotein of hepatitis E virus: delineation of an additional group of positive-strand RNA plant and animal viruses. Proc Natl Acad Sci USA.

[25] Krawczynski K, Bradley DW (1989). Enterically transmitted non-A, non-B hepatitis: identification of virus-associated antigen in experimentally infected cynomolgus macaques. J Infect Dis.

[26] Legrand-Abravanel F, Kamar N, Sandres-Saune K, Garrouste C, Dubois M, Mansuy JM (2010). Characteristics of autochthonous hepatitis E virus infection in solid-organ transplant recipients in France. J Infect Dis.

[27] Legrand-Abravanel F, Kamar N, Sandres-Saune K, Lhomme S, Mansuy J-M, Muscari F (2011). Hepatitis E virus infection without reactivation in solid-organ transplant recipients, France. Emerg Infect Dis.

[28] Lu L, Li C, Hagedorn CH (2006). Phylogenetic analysis of global hepatitis E virus sequences: genetic diversity, subtypes and zoonosis. Rev Med Virol.

[29] Lynch M, O'Flynn N, Cryan B, Hampl H, Opstelten R (1995). Hepatitis E in Ireland. Eur J Clin Microbiol Infect Dis.

[30] Quintana A, Sanchez L, Larralde O, Anderson D (2005). Prevalence of antibodies to hepatitis E virus in residents of a district in Havana, Cuba. J Med Virol.

[31] Rostamzadeh Khameneh Z, Sepehrvand N, Masudi S (2011). Seroprevalence of hepatitis E among Iranian renal transplant recipients. Hepat Mon.

[32] Stefanidis I, Zervou EK, Rizos C, Syrganis C, Patsidis E, Kyriakopoulos G (2004). Hepatitis E virus antibodies in hemodialysis patients: an epidemiological survey in central Greece. Int J Artif Organs.

[33] Sylvan SP (1998). The high rate of antibodies to hepatitis E virus in young, intravenous drug-abusers with acute hepatitis B-virus infection in a Swedish community: a study of hepatitis markers in individuals with intravenously or sexually acquired hepatitis B-virus infection. Scand J Infect Dis.

[34] Takahashi M, Kusakai S, Mizuo H, Suzuki K, Fujimura K, Masuko K (2005). Simultaneous detection of immunoglobulin A (IgA) and IgM antibodies against hepatitis E virus (HEV) is highly specific for diagnosis of acute HEV infection. J Clin Microbiol.

[35] Tang YW, Wang JX, Xu ZY, Guo YF, Qian WH, Xu JX (1991). A serologically confirmed, case-control study, of a large outbreak of hepatitis A in China, associated with consumption of clams. Epidemiol Infect.

[36] Taremi M, Mohammad Alizadeh AH, Ardalan A, Ansari S, Zali MR (2008). Seroprevalence of hepatitis E in Nahavand, Islamic Republic of Iran: a population-based study. East Mediterr Health J.

[37] Tsega E, Krawczynski K, Hansson BG, Nordenfelt E, Negusse Y, Alemu W (1991). Outbreak of acute hepatitis E virus infection among military personnel in northern Ethiopia. J Med Virol.

[38] Yamashita T, Mori Y, Miyazaki N, Cheng RH, Yoshimura M, Unno H (2009). Biological and immunological characteristics of hepatitis E virus-like particles based on the crystal structure. Proc Natl Acad Sci USA.

