# Supplementary material for: The relationship between serology of hepatitis E virus with liver and kidney function in kidney transplant patients
Source: EXCLI J. 2016 Jun 2;15:343–9. doi: 10.17179/excli2016-232 (PMC4928011; doi:10.17179/excli2016-232)
Supplement: Supplementary table 1 [file EXCLI-15-343-s-001.pdf]

EXCLI Journal 2016;15:343-349 – ISSN 1611-2156
Received: March 04, 2016, accepted: May 23, 2016, published: June 02, 2016

Suppplementary material to:

THE RELATIONSHIP BETWEEN SEROLOGY OF HEPATITIS E VIRUS WITH LIVER AND KIDNEY FUNCTION IN KIDNEY TRANSPLANT PATIENTS

Abbas Ali Zeraati[1], Fatemeh Nazemian[1], Ladan Takalloo[1], Amirhossein Sahebkar[2,3], Elahe Heidari[4], Mohammad Ali Yaghoubi[5]*

[1] Kidney transplantation Complications Research Center, Mashhad University of Medical Sciences, Mashhad, Iran
[2] Biotechnology Research Center, Mashhad University of Medical Sciences, Mashhad, Iran
[3] Metabolic Research Centre, Royal Perth Hospital, School of Medicine and Pharmacology, University of Western Australia, Perth, Australia
[4] Department of Pediatrics, Imam Reza Hospital , Mashhad University of Medical Sciences, Mashhad, Iran
[5] Endocrine Research Center, Ghaem Hospital, Mashhad University of Medical Science, Mashhad, Iran
* Correspondence: Mohammad Ali Yaghoubi, yaqubima@yahoo.com

http://dx.doi.org/10.17179/excli2016-232

This is an Open Access article distributed under the terms of the Creative Commons Attribution License (http://creativecommons.org/licenses/by/4.0/).

EXCLI Journal 2016;15:343-349 – ISSN 1611-2156
Received: March 04, 2016, accepted: May 23, 2016, published: June 02, 2016

Patient no	Age (years)	Sex	Type of tra	Duration o	Dialysis typ	History of t	Serum Cr (	Serum Cr (	Serum Cr (	Serum Cr (	Serum AST (	Serum AST	Serum AST	Serum AST	Serum ALT (	Serum ALT	Serum ALT (	Serum ALT (	Serum ALP (	Serum ALP (	Serum ALP (	Serum ALP (U	HEVAb (OD)	
1	23	0	1	14	1	0	1.1	1.2	1.3	1.1	19	20	22	18	44	16	22	30	189	469	150	170	0.32	
2	22	0	0	10	1	1	1.3	1.3	1.2	1.1	34	30	22	20	33	22	24	28	170	180	189	170	0.35	
3	29	0	1	23	1	0	1.2	1.2	1.1	1.2	14	16	18	15	20	18	7	8	114	150	190	160	0.38	
4	33	0	1	21	1	0	1.6	2.4	2.2	1.4	20	14	16	14	18	20	15	6	236	240	200	236	0.32	
5	56	0	0	24	0	0	1.8	1.9	1.7	1.7	19	20	15	14	15	14	15	20	224	230	220	210	1.9	
6	33	1	0	30	1	0	1.8	1.6	1.5	1.4	14	15	20	15	13	18	15	20	240	220	240	210	5.5	
7	47	1	1	18	1	0	2.5	2.5	2	2	16	19	16	16	18	17	20	20	171	170	160	179	0.35	
8	22	1	0	10	1	0	1.1	1.2	1.1	1.1	14	16	18	20	18	16	15	15	227	220	225	220	0.39	
9	19	0	1	17	1	1	2	1.4	1.3	1.3	11	15	15	20	18	20	17	18	230	270	270	437	0.24	
10	33	0	1	18	1	0	1.5	1.2	1.2	1.2	17	22	30	30	12	15	15	20	188	180	190	170	0.25	
11	17	1	1	11	0	0	1.8	1.6	1.4	1.4	21	20	18	15	23	30	32	40	220	200	210	194	0.26	
12	39	0	1	21	1	0	1.2	1.2	1.4	1.4	12	13	20	13	13	10	15	15	157	160	150	150	0.37	
13	24	1	0	12	1	0	1.4	1.2	1.4	1.4	19	20	20	39	13	15	18	27	280	240	230	220	0.29	
14	23	1	1	13	1	1	1.2	1.3	1.2	1.2	12	19	18	19	17	18	17	20	232	220	220	230	0.35	
15	55	0	1	23	1	0	1.3	1.3	1.2	1.2	35	40	42	62	55	50	52	99	240	260	320	327	0.29	
16	33	1	0	12	1	1	1.4	1.4	1.5	1.5	36	32	40	30	18	22	19	20	330	260	280	218	0.3	
17	55	1	0	26	1	0	2.8	2.7	2.6	2.7	16	19	22	15	10	18	15	12	114	120	142	151	3.4	
18	50	1	0	48	1	1	2.1	1.8	1.6	1.3	15	20	17	16	13	18	19	27	440	400	420	400	6	
19	49	1	1	21	1	0	1.8	1.3	1.2	1.2	13	13	15	20	9	12	18	17	251	240	250	260	0.25	
20	23	1	1	10	1	1	2.6	2.5	2.6	2.7	15	15	13	21	12	18	19	22	360	320	380	387	0.25	
21	27	0	0	30	1	0	0.9	0.8	1	0.8	20	18	16	22	13	12	20	18	280	280	270	297	5.8	
22	26	1	1	10	1	0	1.9	1.8	1.9	2.08	29	32	30	33	33	28	25	23	220	290	287	279	6	
23	23	0	1	10	1	1	2.1	1.9	1.7	1.8	30	35	32	40	20	18	15	22	210	220	211	230	0.54	
24	51	1	0	37	1	0	1.9	1.8	2	2.1	19	62	60	58	17	40	80	82	260	280	220	202	1.4	
25	34	1	0	47	1	1	1.6	1.8	1.9	1.9	17	18	20	20	27	22	20	11	111	120	125	153	6.1	
26	61	1	1	34	1	0	2.5	2.2	2.3	2.2	20	32	23	25	25	20	18	22	210	200	220	194	0.3	
27	32	1	0	12	1	0	1.2	1.2	1.3	1.4	19	12	17	11	24	20	18	15	240	243	253	250	0.27	
28	55	0	1	18	1	0	1.3	1.2	1.3	1.4	9	15	12	13	11	13	16	15	150	140	175	147	0.39	
29	32	1	1	24	1	0	1.2	1.1	1.2	1.3	14	17	18	15	29	25	26	27	115	120	110	99	0.34	
30	42	0	0	19	1	1	1.5	1.4	1.2	1.1	18	19	25	20	13	11	17	16	220	230	240	227	0.34	
31	24	0	1	18	1	0	1.7	2	1.9	2.1	16	20	22	15	19	15	18	11	228	220	215	213	0.28	
32	35	0	1	17	1	0	2.1	1.9	1.6	1.6	19	17	20	13	20	15	18	11	242	190	220	198	0.39	
33	31	1	1	23	1	0	2	1.9	1.8	1.8	20	18	30	28	34	38	40	45	518	490	500	540	0.28	
34	49	1	0	30	1	1	1.2	1.2	1.1	1	10	13	15	17	29	35	30	28	235	230	230	240	6.2	
35	24	1	1	14	1	1	1.2	1.1	1.3	1.1	30	32	28	20	34	30	28	34	518	500	512	540	0.3	
36	59	0	0	42	1	0	1.7	1.3	1.1	1.1	24	30	32	27	16	16	20	19	260	251	251	240	0.4	
37	51	1	1	34	1	0	1.2	1.1	1.2	1.3	18	20	15	11	23	27	26	21	240	245	250	210	0.33	
38	34	1	1	21	1	0	1.6	1.9	1.7	2.2	36	32	29	27	44	40	62	74	258	240	230	237	0.42	
39	52	0	1	28	1	1	2.8	2.6	1.7	2.1	30	40	35	30	21	28	20	19	221	230	220	219	0.55	
40	49	1	0	8	1	0	1.3	1.2	1.1	1.1	45	40	30	38	19	17	20	15	280	261	250	260	1.7	
41	42	0	1	13	1	0	1.1	1.3	1.2	1.3	32	28	20	30	18	17	15	20	270	260	270	295	0.27	
42	34	1	1	21	1	0	1.3	1.1	1.2	1.1	19	17	11	21	25	19	22	29	301	320	280	293	0.35	
43	50	1	1	36	1	1	2.1	2	1.9	1.9	27	30	35	28	10	19	20	13	280	290	220	210	0.29	
44	24	0	1	12	1	0	1.2	1.1	0.9	0.8	12	13	12	10	6	20	11	7	463	420	410	390	0.46	
45	53	1	1	37	1	1	1	1.1	1	1.2	19	20	22	18	45	40	48	45	540	580	600	620	6	
46	40	1	1	15	1	0	1.4	1.4	1.3	1.2	53	50	35	27	44	42	45	37	324	300	320	350	0.37	
47	17	0	1	12	0	0	2.3	2.1	2	1.8	11	35	40	27	10	15	12	17	226	280	310	300	0.55	
48	39	1	0	18	1	0	1.3	1.1	1.2	1.2	19	20	18	25	36	40	38	104	250	220	239	248	5.9	
49	53	1	1	31	1	1	1.8	1.9	1.8	1.7	24	25	30	26	9	13	17	35	280	250	280	290	0.35	
50	15	0	0	9	1	0	1.02	1.1	1	0.5	15	15	17	19	12	13	10	12	211	220	210	209	0.32	
51	23	0	1	12	1	0	1.1	1.1	1	1.2	20	18	22	19	30	40	35	38	280	260	240	260	0.38	
52	47	1	1	21	1	0	1.6	1.7	1.5	1.5	22	20	18	20	27	26	30	28	199	210	200	190	0.27	
53	31	1	0	10	1	0	1.9	1.8	1.9	1.7	17	20	22	19	52	60	50	52	157	180	160	165	0.27	
54	30	0	1	13	1	0	2.2	2.1	1.9	1.9	23	20	24	20	15	13	18	17	175	160	150	104	0.45	
55	36	1	1	14	1	0	1.7	1.5	1.3	1.1	28	20	25	26	38	30	35	30	251	245	240	250	0.45	
56	40	0	1	18	1	0	1.7	1.6	1.7	1.7	23	33	40	32	83	115	135	182	511	591	600	655	0.79	
57	26	1	1	12	1	0	1.8	1.7	1.7	1.6	12	10	21	15	13	12	11	15	176	180	170	175	0.32	
58	30	0	1	14	1	1	1.3	1.2	1.1	1.2	38	25	20	24	40	38	45	40	290	280	220	280	0.3	
59	25	1	0	12	1	0	2	1.9	1.9	1.7	14	20	22	24	13	15	10	18	140	170	160	210	0.3	
60	17	0	1	7	1	0	2.6	2.5	2.2	2.3	12	15	9	18	10	15	17	10	210	200	190	210	0.37	
61	28	1	0	48	1	1	1.1	1.2	1.1	1	21	15	17	19	19	18	20	22	136	140	150	145	5.8	
62	56	1	0	30	1	0	1.6	1.4	1.5	1.5	30	28	25	29	15	19	17	15	270	290	280	290	5.6	
63	59	1	1	34	1	1	1.1	1.2	1	1	13	20	18	15	12	15	17	20	160	158	160	150	0.67	
64	19	0	1	8	1	0	1.7	1.5	1.6	1.6	18	17	18	15	20	28	20	22	160	170	180	177	0.3	
65	32	0	1	12	1	1	1.2	1.1	1.1	1.2	12	15	18	17	10	13	12	10	150	155	160	152	0.39	
66	31	0	1	10	1	1	1.1	1.2	1.1	1	20	25	22	23	10	12	15	12	282	290	295	280	0.29	
67	48	1	1	30	1	1	2.2	2.1	2	2	18	17	16	15	19	20	22	13	160	137	130	157	0.35	
68	20	0	0	42	1	1	1.9	1.8	1.6	1.7	26	20	22	24	39	30	32	28	257	260	250	240	1	
69	50	1	1	21	1	1	2	1.9	1.9	1.7	19	22	16	13	24	18	15	12	215	219	240	238	0.5	
70	28	0	0	19	1	0	1.1	1.2	1.1	1	20	28	22	25	21	16	20	19	170	175	180	198	0.24	
71	49	0	0	60	1	1	1.4	1.5	1.4	1.4	28	30	29	25	14	20	19	15	208	210	217	220	6.3	
72	34	0	0	12	1	0	1.2	1.1	1.2	1.1	39	30	32	34	52	45	48	40	180	210	200	215	0.49	
73	43	0	1	11	1	0	1.5	1.3	1.4	1.4	18	19	17	20	15	13	20	19	190	200	170	120	0.4	
74	29	1	1	13	1	1	1.4	1.5	1.4	1.5	40	35	34	39	65	68	60	56	190	180	185	182	0.58	
75	54	1	0	32	0	0	1.9	2.1	2	2.1	20	22	18	16	16	19	23	18	160	155	152	150	6	
76	28	1	1	12	0	0	1.2	1.3	1.2	1.4	36	30	32	31	99	100	110	103	550	560	540	577	0.3	
77	52	1	0	28	0	0	1.6	1.5	1.4	1.3	22	24	22	24	58	77	80	74	410	390	320	381	5.8	
78	51	1	1	31	1	0	1.4	1.5	1.4	1.3	33	30	35	32	28	30	32	29	210	220	200	210	5.8	
79	18	0	1	10	1	1	1.5	1.4	1.3	1.3	32	40	38	35	33	29	32	35	320	341	331	340	0.77	
80	47	1	0	29	1	0	1.3	1.2	1.1	1.2	31	45	40	42	45	17	18	30	260	265	250	262	0.27	
81	37	0	1	18	1	0	1.1	1.2	1.1	1	18	19	17	15	20	18	19	22	201	220	210	210	0.6	
82	27	0	1	12	1	1	2.7	2.9	2.6	2.6	13	15	19	17	11	12	15	12	290	280	270	280	0.46	
83	56	1	1	18	1	1	2.01	1.8	1.7	1.7	13	17	19	15	25	24	28	26	210	230	235	230	5.9	
84	29	0	0	11	1	0	1.2	1.3	1.2	1.1	10	12	17	15	13	11	15	18	121	150	149	140	0.32	
85	45	0	1	30	1	0	2.1	1.9	1.7	1.7	15	22	18	20	15	13	17	37	350	360	355	349	0.3	
86	22	0	1	11	1	0	1.1	1.2	1	1.1	13	11	15	10	22	20	17	20	170	175	160	162	0.39	
87	52	0	1	22	1	0	1.3	1.2	1.2	1.1	18	20	22	19	30	32	29	30	231	240	300	290	0.34	
88	17	0	0	10	0	0	1.5	1.4	1.3	1.4	30	25	29	28	40	39	42	35	222	215	230	210	0.53	
89	45	0	1	28	1	1	2.6	2.5	2.4	2.4	26	27	25	22	22	29	24	23	121	125	120	128	3.14	
90	30	1	0	14	1	0	1.01	1	0.9	1	19	17	20	15	35	32	31	29	130	132	129	121	0.34	
91	26	0	1	12	1	1	1.3	1.2	1.2	1.1	16	19	15	14	12	19	13	17	121	127	126	120	0.5	
92	36	1	1	18	1	0	1.5	1.4	1.4	1.5	149	45	41	39	480	90	112	89	188	210	200	190	0.49	
93	41	0	1	13	1	0	1.1	1.2	1.1	1	14	18	17	15	10	13	19	17	215	220	210	211	0.41	
94	62	0	1	26	1	1	1.3	1.2	1.1	1.2	18	17	14	19	27	32	30	33	268	250	260	270	5.9	
95	50	1	0	28	1	1	1.3	1.2	1.2	1.3	20	18	19	17	32	30	35	33	190	210	200	220	0.32	
96	56	1	0	32	1	0	2.3	2.2	1.3	1.3	38	40	42	44	48	91	95	97	220	230	210	220	5	
97	39	0	1	13	1	0	1.2	0.9	1	1.1	19	20	18	16	1.7	15	16	13	350	340	352	342	0.46	
98	20	1	0	10	1	0	1.1	0.9	0.9	1	12	11	13	14	18	15	17	13	200	210	170	190	0.29	
99	19	1	1	12	1	1	1.9	1.8	1.7	1.7	30	28	25	32	27	32	29	30	346	330	329	356	0.32	
100	37	1	1	24	1	0	1.5	1.4	1.4	1.3	20	19	13	18	23	27	25	24	298	300	290	275	5.8	
101	41	1	1	18	1	1	1.9	1.8	1.7	1.7	27	25	23	24	28	30	27	22	193	170	165	123	0.26	
102	33	0	1	19	1	0	1.5	1.4	1.3	1.2	17	20	19	18	10	19	13	15	153	170	190	165	0.25	
103	39	0	0	21	1	0	1.3	1.2	1.4	1.3	16	19	17	15	76	70	69	72	177	170	165	160	0.4	
104	60	1	1	32	1	0	1.9	1.7	1.8	1.7	35	30	28	32	42	49	55	52	295	282	280	275	0.5	
105	50	1	1	23	1	1	0.6	1.1	1	1.2	26	32	29	27	12	15	13	17	250	260	245	262	0.23	
106	41	1	1	18	1	0	1.9	1.8	1.7	1.5	18	20	19	22	27	32	30	29	196	210	190	187	0.36	
107	53	1	1	21	1	0	1.7	1.6	1.5	1.5	26	29	28	22	25	30	29	26	210	215	230	240	0.58	
108	25	0	1	13	1	0	1.8	1.7	1.6	1.6	19	17	16	15	27	32	30	26	180	179	170	165	0.26	
109	27	0	0	16	1	0	1.6	1.7	1.6	1.6	10	13	17	14	18	19	17	15	129	130	128	125	0.35	
110	28	1	1	15	1	0	1.2	0.9	0.8	1	17	19	18	16	8	13	12	15	192	190	185	180	0.4	
	36.97	N/A	N/A	20.41	N/A	N/A	1.59	1.53	1.46	1.45	22.48	23.00	22.84	22.41	29.07	26.99	28.23	29.64	238.35	241.82	237.61	240.53	1.36	Mean
	12.79	N/A	N/A	10.27	N/A	N/A	0.46	0.44	0.40	0.41	14.82	9.10	8.50	9.20	46.31	19.02	21.83	25.42	92.25	93.28	92.65	102.39	2.05	SD

Abbreviations

Cr	Creatinine
AST	Aspartate aminotransferase
ALT	Alanine aminotransferase
ALP	Alkaline phosphatase
HEVAb	Hepatitis E virus antibody
OD	Optical density

Code identifiers

Sex	0 (female), 1 (male)
Type of trar	0 (living), 1 (cadaveric)
Dialysis typ	0 (peritoneal dialysis), 1 (hemodialysis)
History of tr	0 (no), 1 (yes)
